# Watch and Learn: Seeing Is Better than Doing when Acquiring Consecutive Motor Tasks

**DOI:** 10.1371/journal.pone.0038938

**Published:** 2012-06-18

**Authors:** Beverley C. Larssen, Nicole T. Ong, Nicola J. Hodges

**Affiliations:** School of Kinesiology, University of British Columbia, Vancouver, British Columbia, Canada; Centre national de la recherche scientifique, France

## Abstract

During motor adaptation learning, consecutive physical practice of two different tasks compromises the retention of the first. However, there is evidence that observational practice, while still effectively aiding acquisition, will not lead to interference and hence prove to be a better practice method. Observers and Actors practised in a clockwise (Task A) followed by a counterclockwise (Task B) visually rotated environment, and retention was immediately assessed. An Observe-all and Act-all group were compared to two groups who both physically practised Task A, but then only observed (ObsB) or did not see or practice Task B (NoB). The two observer groups and the NoB control group better retained Task A than Actors, although importantly only the observer groups learnt Task B. RT data and explicit awareness of the rotation suggested that the observers had acquired their respective tasks in a more strategic manner than Actor and Control groups. We conclude that observational practice benefits learning of multiple tasks more than physical practice due to the lack of updating of implicit, internal models for aiming in the former.

## Introduction

Time spent in physical practice provides individuals with the direct means to compare their motor commands to movement output (sensory consequences). This type of practice allows the implicit development and updating of internal models that aim to create congruency between motor commands and outcome goals [1], [2]. An internal model is typically defined as a context-dependent, neural representation of a specific motor task that specifies appropriate patterns of muscle activation to facilitate accurate movements within a given environment (e.g., [1]). Motor learning results in the updating or acquisition of an internal model for a practised motor task.

Physical practice is typically presented as the best method for the successful acquisition of motor skills and even though benefits are seen as a result of other types of practice, such as observation and imagery, benefits do not exceed those of physical practice (for observational learning reviews see [3], [4], [5]). Therefore, it is of significant interest if benefits for observational over physical practice can be shown for the acquisition of sensory-motor skills.

Demonstration of a skill provides us with a useful guide for our actions [6]. From demonstrations, individuals have learned explicit strategies that they can employ when physical performance is required (e.g., [7]). It has also been suggested that observational practice techniques might also work to aid motor skill acquisition through a more motor-based matching process via what has been termed a “mirror neuron system” in the premotor cortex (e.g., [8], [9]). There is thought to be functional equivalence between acting and mental operations involved in action-observation and imagery, such that mental simulation of a movement is thought to lead to similar (albeit sub-threshold) patterns of muscle activation compared to actual physical execution of the desired movement [10].

In support of this motor-driven process, Mattar and Gribble [11] showed that observers could learn how to respond in a pointing task to mechanical force-field perturbations applied to an actor. They suggested that neural representations (internal models) could be acquired visually by observers who were naïve to the task. Control experiments showed that observational learning was interfered with by a secondary motor task but not a verbal task, supporting their suggestion that observational learning occurred via more implicit, motor-driven means (see also [12], [13], [14]).

Despite these positive learning effects and the suggestion that observational practice processes are similar to physical practice, there is reason to be cautious in accepting this conclusion. There is little evidence to support the involvement of mirror neuron activation during observation of tasks that have not been practised [14]. A lack of explicit knowledge or increased interference from a more cognitive task in comparison to a more motoric secondary task does not necessarily imply implicit learning and/or motor-related activation. Further, evidence of implicit motor learning has been assumed based on a lack of transfer across effectors following observational practice [12]. However, this result has not been replicated in other sequence learning tasks (e.g., [15]), implying the development of a more visual-spatial rather than motor representation. In addition, effector transfer has been shown in adaptation tasks irrespective of the level of awareness developed during physical practice [16]. Perhaps the most important result that speaks against a more implicit, motor-based representation as a function of observational practice has been the absence of after-effects following experience in a novel adapted environment. The presence of after-effects has been taken as evidence that an implicit, internal model has been acquired or updated as the practice experience in one environment leads to immediate, unintentional errorful performance, when transferred back to a normal environment (e.g., [17], [18], [19]). Ong and Hodges ([20], [21]) conducted a visuomotor adaptation study, where observers were shown videos of an actor learning to aim in a clockwise rotated environment, signified by a discrepancy between the hand movements of the actor and the resulting cursor trajectory. Like participants from Mattar and Gribble’s study [11], the observers learnt through observation as evidenced by significant savings when first physically exposed to the watched rotated environment. However, when tested in a known normal environment immediately following observation, observers showed no signs of negative after-effects. This was in contrast to two physical practice groups, who practised with either vision of the rotated cursor or both vision of the cursor and their hand [20]. These findings of direct learning effects in the absence of after-effects were replicated in a second study [21] where a mixed observation and physical practice group was also studied and continual probes of explicit awareness were taken during practice. These two studies led to the conclusion that observation and physical practice operate by different learning mechanisms, with physical practice necessary for the acquisition of implicit, motor-based representations that lead to updating of internal models.

A second paradigm has been used to study these adaptive processes whereby learning is assessed through measures of interference across acquisition of multiple motor skills. Interference can be either retrograde: the introduction and practice of a novel task interferes with recall of the preceding activity, or anterograde: a previously learned task interferes with the acquisition of a subsequent task [22]. When presented with the task of adapting consecutively to two opposing, rotationally perturbed pointing tasks, Krakauer et al. [19] observed retrograde interference of the second adaptation task (Task B) on the retention of the first (Task A). This suggested that only one internal model can be retained or consolidated at a time [23].

Based on the absence of after-effects and the hypothesis that observational practice does not result in an updating of a previous internal model [20], in the current experiment we tested how observation or physical practice of a second task (counterclockwise rotation; Task B) following either observation or physical practice of a first task (clockwise rotation; Task A) interferes with memory (performance) of Task A. In the only study where concurrent learning of different skills following observation has been examined (designed to study memory consolidation), Trempe, Sabourin, Rohbanfard, and Proteau [24] found that regardless of the time interval between the observation of two different sequence-timing tasks, observation of sequence B did not interfere with sequence A (i.e., no retrograde interference). However, opposite to previous research, a similar lack of interference was also shown for a physical practice group suggestive of unique task-features. In a subsequent experiment with the same task, different patterns of interference as a result of observing and doing following a short or long break were found, leading the authors to speculate that observational learning relied more on declarative “explicit” learning processes as opposed to physical practice that relied on more procedural “motor” memory processes.

Based on previous data and these hypotheses we expected that observational practice would be characterized by more explicit awareness of the tasize and direction of the rotations as well as longer response times (RTs), indicative of greater involvement of working memory and strategically-driven processes [25], [26]. RT measures were also expected to provide a secondary measure of interference associated with practising two tasks back to back and subsequently attempting to recall and execute either of these tasks. Therefore, slower RTs were expected for all groups who had practised (or observed) two tasks (A and B) in comparison to a no Task B control group.

We predicted that both Actor and Observer groups would learn through seeing and doing by showing improved retention on practised tasks compared to a no-practice control group. Importantly, if only acting results in the updating of internal models, only a group who physically practices both Tasks A and B would show a significant increase in error when retested on Task A in comparison to their performance at the end of initial practice on Task A. A no-Task B control group and an observer group that observes Task B after physical practice of A, would not show the same increase in errors, indicative of this interference associated with the difficulty in holding two opposing internal models simultaneously. We also expected that errors on Task A would be higher for an Actor group who physically practises both tasks in comparison to an observer group who only observes both tasks. We do expect some interference as a result of practising two tasks and being able to recall what actions are needed to perform in both environments, but the degree of interference was expected to be significantly less in observers compared to actors. In summary, we hoped to show that two similar motor tasks can be learned and retained when presented in close juxtaposition, if at least one is learned through observation.

## Results

### Mean Directional CE

#### Pre-test

**Figure 1:**
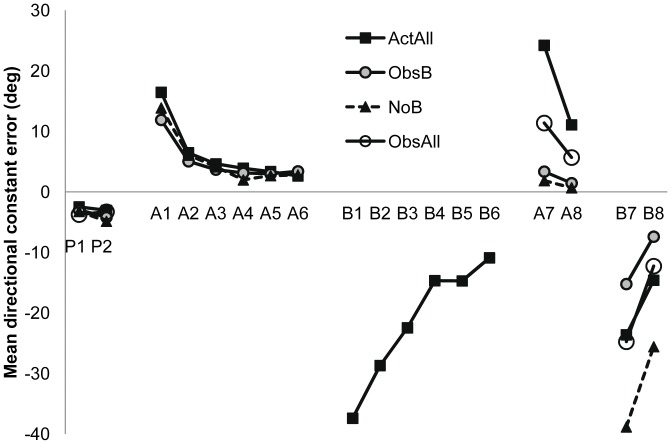
Performance error as a function of experimental group and practice phase. Mean directional constant error (degrees) as a function of block for the ActAll, ObsB, ObsAll and NoB groups in normal environment pre-tests (P1, P2), across physical practice of Task A (clockwise rotation, A1–A6) and Task B (counterclockwise rotation, B1–B6) and in tests of retention of Tasks A (A7, A8) and B (B7, B8).

The groups were not different from each other, F<1 (see Figure 1). Errors showed a reliable but small increase across blocks 1 (−2.93) and 2 (−3.77), F(1,28)  = 5.83, *p*  = .02, *η_p_^2^* = .17.

#### Practice

The ActAll, Control and ObsB groups all received the same physical practice of Task A and hence there were no group differences, F<1. All groups decreased errors across blocks in a linear fashion, F(1,21)  = 119.41, *p*<.001, *η_p_^2^* = .85 (see Figure 1). Errors decreased from 14.42° in block 1 to 2.79° in block 6. There was no interaction, F  = 1.

Only the ActAll group physically practised Task B. Comparisons across Task A and B for this group yielded a significant task, F(1,7)  = 88.03, p<.001, *η_p_^2^* = .93 and block effect, F(1.85, 12.98)  = 6.90, *p* = .01, *η_p_^2^* = .50, as well as an interaction, F(1.80, 12.61)  = 39.13, *p*<.001, *η_p_^2^* = .85. As can be seen in Figure 1, errors decreased across blocks in a similar fashion for both tasks, but errors for Task B were significantly higher than for Task A for blocks 1–3.

To determine whether errors on Task B (i.e., CCW rotation) were a function of task difficulty associated with Task B, and/or anterograde interference of Task A (CW) on Task B, we tested 7 individuals just on Task B (again following a 50 trial no-rotation pre-test). The mean unsigned errors were similar to those reported for the ActAll group for Task A (B1 = 18.17, B2 = 8.44… B5 = 4.02, B6 = 2.69°). A statistical comparison of Task A (across the 6 blocks of practice) for the ActAll group, to the new Task B only group, yielded F values close to 1 (group, F = 1.01, Group x Block, F  = 1.12), suggesting that Task A and B did not differ in nominal task difficulty.

#### Retention

These data are plotted on the right of Figure 1. As predicted, the ActAll group showed the highest error when tested for retention of Task A. This was evidenced by a significant group effect, F(3,28)  = 21.05, *p*<.001, *η_p_^2^* = .69. As predicted, the ActAll group showed more errors than the 3 other groups (*p*s<.01). Although the two observer groups were not different from each other, the ObsAll group had higher error than the NoB control group (*p*<.05). The block, F(1,28)  = 92.38, *p*<.001, *η_p_^2^* = .77 and Group x Block effects, F(3,28)  = 27.26, *p*<.001, *η_p_^2^* = .75 were significant due to a reduction in errors across blocks, particularly for the ActAll group, although this group maintained the highest error even in block 2.

Comparisons of the last 2 blocks of practice to the retention tests for the ActAll, ObsB and NoB groups yielded a Group x Phase interaction, F(2,21)  = 85.50, *p*<.001, *η_p_^2^* = .89. Only the ActAll group showed a significant increase in error from practice (A5, A6) to retention testing (A7, A8, Figure 1).

Importantly, the observer groups had learnt from watching, evidenced by a significant group effect for Task B, F(3,28)  = 8.96, *p*<.001, *η_p_^2^* = .49. As shown in Figure 1, the observer groups were not significantly different to the ActAll group (who physically practised Task B), but all groups were more accurate than the NoB, control (who did not see or practice Task B, all *p*s<.05). A block effect, F(1,28)  = 113.85, *p*<.001, *η_p_^2^* = .80, was due to a decrease in errors across blocks, but there was no interaction, F(3,28)  = 2.59, *p* = .07, *η_p_^2^* = .22, 1−*ß*  = .57. Only the ActAll group had practice data for Task B and as with Task A, this group showed a significant increase in the magnitude of errors across the two phases (last 2 blocks of adaptation practice and retention), F(1,7)  = 89.03, *p*<.001, *η_p_^2^* = .93 (mean of B5 & B6 =  −12.81, *SD*  = 7.58° vs. mean of B7 & B8 =  −19.11, *SD*  = 8.25°).

### RTs

#### Pre-test

There were no group differences, F<1 (see Table 1).

#### Retention

Three of the groups, with the exception of the ObsAll group, had physically practiced Task A. Therefore, we expected RTs to be slowest for the ObsAll group, indicative of a more strategic-mode of performance. Indeed, there was a significant group effect, F(3,32)  = 5.52, *p*<.01, *η_p_^2^* = .37. The ObsAll (*M*  = 456 ms) was the slowest, although this group was only significantly slower than the NoB control group (*M*  = 294 ms). The ActAll group (*M*  = 418 ms) was also slower than the NoB control group, suggesting that back to back practice of two tasks leads to interference in recall of the first, as evidenced by slower RTs. The group who alternated between two modes of practice, the ObsB group, did not differ significantly from any group (*M*  = 365 ms).

The groups also differed in their RTs for Task B, F(3,32)  = 4.00, *p*  = .018, *η_p_^2^* = .30. The two observer groups (ObsB, *M*  = 500 ms; ObsAll, *M*  = 565 ms) showed slower RTs than the ActAll (*M*  = 413 ms) and NoB control (*M*  = 417 ms) groups, although only the ObsAll group was significantly slower (*p*s<.05).

**Table 1:** Reaction time data.

Group	Pretest	Retention Task A	Retention Task B
ActAll	354.80 (41.33)	417.99 (100.61)	413.02 (117.39)
ObsB	358.70 (70.66)	364.52 (75.89)	500.35 (116.81)
ObsAll	387.49 (77.26)	455.65 (108.19)	564.96 (98.85)
NoB	340.04 (43.93)	294.47 (28.15)	417.12 (75.54)

Mean reaction times (ms, and between subject SDs) as a function of group and condition (pre-test and retention of Task A and B).

### Strategic Memory and Awareness

These data are displayed in Table 2. What is notable is the poor performance of the NoB, control group for Task A. This group only physically practised Task A and as expected it demonstrated poor memory/awareness of the direction of rotation for this task (*n* = 4 reported that they were unable to tell us anything about the rotation and were unable to complete the diagram). Of the remaining 3 groups, approximately half the participants in each group consistently and correctly determined the correct direction of the rotation for all 5 targets. Despite some evidence that the observer groups were generally more accurate in judging the size of the rotation than the actor group (approximately 19° error (30°–11°) vs 27° error (30°–3°) respectively), all groups underestimated the size of the rotation and there were no group effects, F(3,28)  = 1.45, *p*  = .25, *η_p_^2^* = .15,1−*ß*  = .34.

**Table 2:** Self-report data of task-specific explicit awareness.

	Task A (+30°, clockwise)			Task B (−30°, counterclockwise)		
Group	Dir(n)	Size (M)	Size (SD)	Dir(n)	Size (M)	Size (SD)
ActAll	4	2.71 (25.11)	9.07 (6.46)	3	−7.48 (16.18)	8.26(6.87)
ObsB	5	10.68 (9.28)	3.34 (5.10)	8	−26.05 (9.86)	5.27 (2.47)
ObsAll	5	10.69 (12.61)	8.17 (8.36)	3	−10.18 (16.95)	7.35 (6.49)
NoB	0	−7.55 (10.17)	9.20 (4.81)	–	–	–

Number of participants (out of 8) who consistently reported (on schematic diagrams of the target display) the correct direction (Dir) of the target rotation for all 5 targets, for Task A and B, along with the mean measured size of the rotation from the diagrams for all participants who completed the task (°) and between-target SDs (°) across the 5 targets (between-subject SDs).

For Task B, both observer groups watched this rotation, the ActAll group physically practised and the control participants rested. All of the ObsB (mixed practice) group were consistently correct (i.e., for all 5 targets) in judging the direction of error. In contrast, only 3 participants were correct in the other two groups who received the same mode of practice throughout (i.e., all physical practice or all observation). When we measured the size of the errors, all participants underestimated, but the group effect was significant, F(2,24)  = 3.74, *p*  = .041, *η_p_^2^* = .26, 1−*ß*  = .62 due to greater accuracy of the ObsB group compared to the ActAll group (*p*<.05).

## Discussion

Consecutive physical practice of two different sensory-motor tasks has caused interference in retention of the first task (e.g., [23]). This is thought to be due to difficulties associated with simultaneously holding two (opposing) internal models of the environment. Based on previous work showing a lack of after-effects from watching, despite significant learning benefits [20], [21], we hypothesized that observational practice of these types of tasks would not produce this type of interference, yet still result in successful acquisition. Our hypotheses were confirmed. Different to actors, a group that only observed a second task, following physical practice of a first, did not show evidence of significant interference during retention testing of the first task (and did not differ from a control group who only practised Task A). Comparisons of all groups during retention testing of A, showed significantly higher errors for the Actor group in comparison to two observer groups (ObsB, ObsAll) and the NoB control group. Importantly, observers had learnt Task B from watching. They performed more accurately than a no-Task B control group and as accurately as the actors. The increase in errors going from physical practice of Task A to physical practice of Task B, also shows an anterograde pattern of interference for the actor group (confirmed by comparisons of errors to a Task B only group). In summary, these data suggest that observation allows for a different type of movement representation from that developed from physical practice that is not subject to the same type of between-task interference.

In order to ascertain the potential mechanisms underpinning these results we asked people to report on the remembered size and direction of the target rotation in the two tasks and analyzed RTs. This latter measure provides an index of planning time and slower RTs are assumed to reflect a more explicitly mediated mode of performing [25], [26]. We expected that RTs in the retention task would also provide a secondary measure of interference, particularly of Task B on recall of Task A. That is, RTs were expected to be slower when participants were trying to explicitly determine what to do as a result of negative carry-over effects from practice at a previous task (i.e., retrograde interference).

The observer groups, who had only observed Task B, were generally slower (∼100–150 ms) in initiating their movements during retention of Task B than the Control (NoB) and ActAll groups. Moreover, in terms of awareness and memory of the perturbation for Task B, all ObsB participants correctly and consistently recalled the direction of the rotation for all 5 targets and were more accurate than actors at judging the size of the rotation. However, the ObsAll and ActAll groups were less accurate at recalling the direction of rotation, potentially suggesting some interference from practising both tasks with the same type of practice (all observation or all physical practice), rather than necessarily a less explicit/strategic mode of learning for the observers (see [27]). This hypothesis is further supported by the fact that the ObsAll group showed the slowest RTs for retention of Task A, suggestive of additional interference as a result of learning both tasks through observation, rather than just Task B (although the two observer groups were not significantly different). The ActAll group also showed slower RTs for Task A, in comparison to control participants. This also supports our hypothesis that the slow RTs for Task A were somewhat a result of interference from previous physical or observational practice of Task B.

In terms of explicit awareness of the size and direction of the perturbation during Task A, the NoB, control and ActAll groups showed little indication that they were aware of the strategy required to aim accurately to the target (although significant differences were only noted for the NoB control group). We did, however, only test for explicit knowledge after all the practice and retention phases were complete and as such we have to infer whether these measures were indicative of the processes engaged during practice and/or the development of awareness associated with the need to perform (and recall what to do) in different environments. Because participants in the ObsB group had better (though not significantly better) awareness of the size of the rotation of Task A than the ActAll group, even though both groups had only physically practised this task, awareness could potentially have developed retroactively, after observational experience of Task B. In view of these data and limitations, we do not wish to stress the importance or otherwise of strategic processes in acquiring and performing these tasks. Indeed, we believe that it is not the acquisition of explicit knowledge that matters in terms of behavioural effects; specifically after-effects and interference, but rather the development, or lack of development of implicit, internal models. Similar conclusions have been made by Wang et al. [16] who found that explicit awareness did not moderate transfer effects across unpractised limbs in a visuomotor adaptation task.

In other research conducted in our laboratory, where only one type of adaptation practice was required, we have shown that in general actors are poor at verbalizing strategies for performing and observers show more explicit awareness of the rotations than actors [20], [21]. These differences are apparent even when continual probes of explicit awareness are administered throughout the practice period [21]. Generally observers become more (explicitly) accurate with practice, but actors less accurate (especially in the first few blocks, see also [28]). These data suggest that for actors, the process of learning is generally more implicit. However, there is variability in the degrees of explicit knowledge demonstrated by actors, particularly if vision of the hand is provided. Underscoring our point above, awareness is not related to the presence or absence of after-effects although it does seem to moderate the size of the effects [20]. Therefore, rather than explicit knowledge necessarily protecting against interference effects as a result of back-to-back practice of two different tasks, it appears to be the absence of a more implicit type of learning that explains these interference effects and the differences between acting and observing. Arguably, this implicit learning and the resultant updating of internal models is a result of physically experiencing and adapting to felt hand position and rotated visual target position. The physical experience might be related to the sending of motor commands and subsequent prediction of sensory consequences (i.e., feedforward processes) or feedback processes associated with proprioception. Because a deafferented person can adapt and show after-effects following physical practice with only vision [29], [30], we expect it is more likely a result of efferent-related processes. However, it is also possible that re-afference (i.e., self-generated feedback) is needed to stimulate implicit learning and updating of internal models. Indeed, delaying of this self-generated feedback was sufficient to remove potential after-effects in an arm-wrist visuomotor adaptation experiment, despite feedforward adaptation learning in the rotated environment [31]. Although it has been implied that observation and physical practice are qualitatively similar processes with differences being attributable to the absence of a final movement in the former (e.g., [10], [14]), our data suggest otherwise. There does not seem to be any strong evidence to suggest that motor-related processes are activated during observational practice of a novel task that requires the learning of a new relationship between motor output and visual input. Similar conclusions about the role of more strategic or visually-based, rather than motor-based, representations governing observational practice have been made for the learning of single and dual limb coordination tasks (e.g., [32], [33]) as well as in sequence reproduction tasks (e.g., [15]). It is of course possible that these effects are a product of these types of more visually-based tasks, even though generally dynamic (i.e., force-field based) and visuomotor adaptation tasks have produced similar patterns of after-effects and interference [34], [35].

If it is true that physical practice and observational practice operate by different mechanisms (with the former being more implicit and the latter missing this implicit, motor-based component), this would explain why we see negligible interference on Task A performance when interrupted by trials of observing the Task B counter-rotation. That is, the mechanisms required to learn Task A, are different from those of Task B for the observers, allowing these two opposing rotations to be learnt and retained, without interference from each other. Trempe et al. [24] have also provided evidence that observational practice operates differently to physical practice with respect to offline learning and processes associated with memory consolidation, with the former not being subject to the same types of interference as seen for physical practice. These authors suggest that different declarative (explicit) and procedural (implicit) cortical networks may be involved in learning through observation, with declarative type memories being more involved in observational practice. Increased activation in parietal and prefrontal cortex during explicit learning has been shown [36] and this more explicit process has also been linked to visual-spatial attention/spatial working memory [37] which might also typify observational learning. These observational practice benefits are also more than just a result of practice across different contexts, although they do seem to be moderated by it. Krakauer and colleagues [38] showed that experience of different contexts, particularly with respect to the effector used, can moderate interference and after-effects. They argued that the learning and recall of different rotations can be explained by contextual effects, rather than explicit-implicit distinctions, with each isolated effector or context having its own cue. Because the ObsAll group practiced with the same medium throughout (like the ActAll group), yet was more accurate than the ActAll group in retention of Task A, this suggests that observational practice benefits are more than context effects. However, the ObsAll group showed more evidence of interference than the ObsB group, with respect to their initial physical performance/retention on TaskA, suggesting a moderating effect of context.

In conclusion, physical practice of a second task exerted significant interference effects on retention of a first, previously learnt task, providing further support for the idea that two implicit, internal models cannot be retained well through consecutive bouts of short-term physical practice [23]. As predicted, observational practice, either following physical or observational practice of the opposing rotation, was not subject to this level of interference across the retention interval and observational exposure to the second task. This was despite the fact that observers had learnt this second task. These data add support to the suggestion that observation and physical practice operate via different mechanisms with the former showing less competition during memory consolidation. Physical practice leads primarily to the development of a motor-based representation that is akin to an internal model for performing a specific motor task. This type of practice operates via mostly, though not exclusively, implicit processes [39]. In contrast, observational practice appears to operate by more explicit, strategically-mediated processes and it does not result in the acquisition or updating of implicit, internal models. Potential benefits of observational practice therefore include the absence of after-effects, the ability to retain more than one model or representation of the world concurrently and faster learning (e.g., [40]). However, potential drawbacks of this method of practice, is that it can lead to more effortful recall than physical practice, as inferred from slower RTs, and it may be less robust to interference from time, pressure or secondary tasks than physical practice, which awaits further testing.

## Methods

### Ethics Statement

All procedures were conducted according to the regulations of the Behavioural Research Ethics’ Board of the University of British Columbia who specifically approved this study. Written informed consent was obtained from all participants.

### Participants and Groups

Thirty-two, right-hand dominant participants were pseudo-randomly assigned to four groups (n  = 8/group). Two Observer groups; Observe Task A and B (ObsAll, *M* age = 23.0 yr, *SD* = 2.0, F  = 6) or Observe Task B following physical practice of Task A (ObsB, *M* age = 20.6 yr, *SD* = 1.51, F  = 3); an Actor group that physically practised both Tasks A and B (ActAll, *M* age = 20.6 yr, *SD* = 1.06, F  = 5) and a Control group, that only practiced Task A (NoB control, *M* age = 23.13 yr, *SD* = 2.0, F  = 4) (see Table 3). Actors were assigned first so they could be filmed and yoked to observers. Remuneration of $8/hour was paid to individuals. All participants were self-reported right-hand dominant.

**Table 3:** Experimental groups and practice conditions.

	Pre-test	Adapt/Task		Retention/Task	
Environment	Normal	A (CW)	B (CCW)	A (CW)	B (CCW)
Group/Trial	t = 50	t = 150	t = 150	t = 50	t = 50
ActAll	Act	Act	Act	Act	Act
ObsB	Act	Act	Watch	Act	Act
ObsAll	Act	Watch	Watch	Act	Act
NoB	Act	Act	None	Act	Act

Experimental Groups (ActAll, physically practised A & B; ObsB  =  practised A, observed B; ObsAll  =  observed A & B; NoB  =  Practised A) and Practice Conditions (CW  =  clockwise; CCW  =  counterclockwise).

### Task and Apparatus

Task, apparatus and procedures were similar to those reported in Ong and Hodges [20], [21]. Key aspects and major differences are detailed below. Participants wore a black wrist brace on their right hand and orange rubber finger protector on their right index finger to standardize the appearance of the finger in the observer videos. All participants were tested alone. They sat in a chair facing a virtual environment set-up positioned on a desk, whereby images from an upturned computer monitor (target stimuli or videos) were projected down onto a semi-silvered mirror. Movements were made using a custom mouse on a graphics’ tablet positioned under the mirror (Calcomp Drawing Board VI, 200 Hz, 200 lines/cm resolution) that measured 2D position. Participants covered the mouse with their right hand with their index finger pointing in the direction that they wanted to move. Accuracy was calibrated to the position of the index finger. The room was blacked out and a chin rest placed in front of the set-up ensured consistent vision for all groups and conditions.

The upturned monitor projected an image of the visual stimuli; a central starting red square (0.5 cm inner length) and 5 green targets equidistant from the start square (10 cm) and each other, separated by 72°, and the trajectory of the cursor onto the mirror. The cursor was controlled by the movement of the mouse. All 5 targets were presented randomly in one 5 trial cycle. Participants aimed to targets with their right index finger by sliding the mouse through the target as fast as possible in a straight trajectory (the target turned red for movement times >250 ms). They were told not to stop at the target, but to make a fast, smooth movement through the desired target. Within a cycle, the actor returned to the start square before another target was presented. Vision of the cursor was occluded on return until the participant was within 4 cm of the start square.

During adaptation practice (Task A and Task B), actors saw the visual targets and the rotated cursor trajectory. The observers watched an edited video (filmed via a web camera, Logitech Quickcam Pro 9000) of actors adapting to the rotated environment with the rotated cursor trajectory. A fluorescent light was used to illuminate the actor’s hand for making the videos, but a black-board prevented the actor from seeing their own hand.

### Procedure

The experiment was divided into three phases; Pretest, Adaptation (Task A, 30° clockwise and Task B, 30° counterclockwise rotation), and Retention (Task A and B) (see Table 3). This procedure is different from our previous experiments (e.g., [20], [21]), where no second adaptation environment (Task B) has been introduced. Participants first familiarized to the task under normal conditions (i.e., no rotation) where sight of both target and cursor trajectory was provided. There then followed a 50 trial ‘normal’ pretest in the absence of cursor vision; to assess proprioceptively guided reaching and to ensure the groups were matched before practice (as determined after the experiment by pre-test errors). Vision of the cursor returned once the participant was within 4 cm of the start position. Two consecutive adaptation phases of 150 trials then commenced.

For Task A, the ActAll, ObsB and NoB control groups physically practiced after being told that they would move in an altered environment. Only the ActAll group physically practiced Task B. The Observer groups were told that they would be watching a person learning to aim to targets in an altered environment and that they would be later tested in this (or these) environment(s). Each task was separated by approximately 5 minutes. Both observer groups watched a video of either Task B (ObsB) or both Tasks (ObsAll) and the NoB control group verbally answered questions about handedness for ∼10 minutes in lieu of performing or seeing Task B.

All Groups completed two immediate retention tests; 50 trials of Task A followed by 50 trials of Task B. This order was chosen because our primary hypothesis was with respect to retention of Task A, to determine if observation of an opposing rotation interferes with retention of a previously practised (watched or physically practised) task. During retention testing all participants underwent equivalent conditions involving physically moving to targets with only vision of the rotated cursor trajectory. They were told only that they would again be performing in a different environment, but they were not given any reminders or retention cues and if participants asked or noted similarities they were prompted to continue trying to get the cursor to the target.

After experimental testing, explicit recall or awareness of Task A and B adaptation conditions were assessed. Simple paper schematics of the target display were constructed showing the position of the 5 targets relative to the centre home position. Using a pen and ruler, participants were asked to draw for each of the 5 targets, the approximate finger/hand trajectory required to aim accurately to the targets. This provided us with information about the direction of the movement (i.e., was the hand trajectory to the left or right of the target, indicative of a CW or CCW rotation), as well as the magnitude of this difference from the target (measured in degrees with a protractor). Observers were asked to draw the path of the actor’s finger in the video for Task B (and Task A for the ObsAll group). If participants drew the same trajectory rotation (i.e., direction) for all 5 targets they were judged consistent and if this was in the correct direction they were judged both consistent and correct.

### Analyses

Mean directional constant error (CE) was computed for each cycle of trials. Data collection, filtering and derivation of kinematic information were identical to earlier procedures (e.g., [20]). Specifically, the angle from the origin (home position) to the position of the cursor at peak tangential velocity was computed and this value was subtracted from the intended trajectory angle (i.e., 0, 30 or −30° from the radial target location) to give directional error. Peak velocity occurred at approximately 75% of the distance to the target (group mean ranges across all blocks and conditions  = 71%–81%. There were no significant group differences across any condition). A positive or negative value for error denoted a CW or CCW error respectively. Movement trials that exceeded 300 ms were excluded from analyses (*M_excluded trials_*  = 1.46%, *SD*  = 1.46%; ActAll  = 1.08%, ObsAll  = 2.58%, ObsB  = 1.25%, NoB  = 0.92%). Although we chose to focus on errors at peak velocity, due to the shooting type of movement required, we could have chosen any point along the trajectory. Indeed, analyses of errors at 25%, 50% and 100% of the distance to the target yielded the same pattern of results as those reported below for peak velocity. RTs were calculated for the pre-test and retention tests based on the time between target onset and movement initiation (i.e., when the cursor was more than 0.25 cm from the origin).

Mean directional constant error (CE), average RTs and the mean reported perturbation size, based on post-assessment of explicit knowledge, were analyzed using mixed-factor ANOVAs. Group (Act, ObsAll, ObsB and NoB) was the between-factor and Block (each block consisted of 5 cycles/25 trials), Task (A or B) or Phase (last two blocks of adaptation practice in comparison to retention) were the within-factors. Separate analysis of Task A and Task B were undertaken to facilitate understanding of practice and retention effects and to accommodate for the different conditions of practice associated with the various groups. Partial eta squared (*η^2^_p_* ) values are reported for effect size, post hoc analyses were conducted using Tukey HSD (*p*<0.05) and power calculations are reported (1−*ß*) for non-significant effects (F>1).
